# Synthesis and electrochemical performance of Li_2_Co_1−_*_x_*M*_x_*PO_4_F (M = Fe, Mn) cathode materials

**DOI:** 10.3762/bjnano.4.97

**Published:** 2013-12-09

**Authors:** Nellie R Khasanova, Oleg A Drozhzhin, Stanislav S Fedotov, Darya A Storozhilova, Rodion V Panin, Evgeny V Antipov

**Affiliations:** 1 Department of Chemistry, Moscow State University, Moscow 119991, Russia

**Keywords:** energy related, fluorophosphates, high-energy cathode materials, high-voltage electrolyte, Li-ion batteries, nanomaterials, reversible capacity

## Abstract

In the search for high-energy materials, novel 3D-fluorophosphates, Li_2_Co_1−_*_x_*Fe*_x_*PO_4_F and Li_2_Co_1−_*_x_*Mn*_x_*PO_4_F, have been synthesized. X-ray diffraction and scanning electron microscopy have been applied to analyze the structural and morphological features of the prepared materials. Both systems, Li_2_Co_1−_*_x_*Fe*_x_*PO_4_F and Li_2_Co_1−_*_x_*Mn*_x_*PO_4_F, exhibited narrow ranges of solid solutions: *x* ≤ 0.3 and *x* ≤ 0.1, respectively. The Li_2_Co_0.9_Mn_0.1_PO_4_F material demonstrated a reversible electrochemical performance with an initial discharge capacity of 75 mA·h·g^−1^ (current rate of C/5) upon cycling between 2.5 and 5.5 V in 1 M LiBF_4_/TMS electrolyte. Galvanostatic measurements along with cyclic voltammetry supported a single-phase de/intercalation mechanism in the Li_2_Co_0.9_Mn_0.1_PO_4_F material.

## Introduction

In recent years the range of application of Li-ion batteries has been expanded from small-sized portable electronics to large-scale electric vehicles and stationary energy storage systems. Large-scale energy applications require batteries that are economically efficient, highly safe and that provide a high energy and power density. Today most of the cells in use have almost reached their intrinsic limits, and no significant improvements are expected. Therefore, current research in this field is directed towards the development of new high-performance materials. The specific energy of Li-ion batteries can be enhanced by applying cathode materials that operate at high voltages, and/or by increasing the specific capacity with materials that could cycle more than one Li atom per active transition metal atom. In this respect, fluorophosphates of the general formula A_2_MPO_4_F seem to be very attractive since they are expected to exhibit a high operating potential because of the increased ionicity of the M–F bond. Furthermore, A_2_MPO_4_F cathode materials may reach capacity values larger than 200 mA·h·g^−1^, if more than one lithium atom would participate in the reversible de/intercalation process.

**Figure 1:**
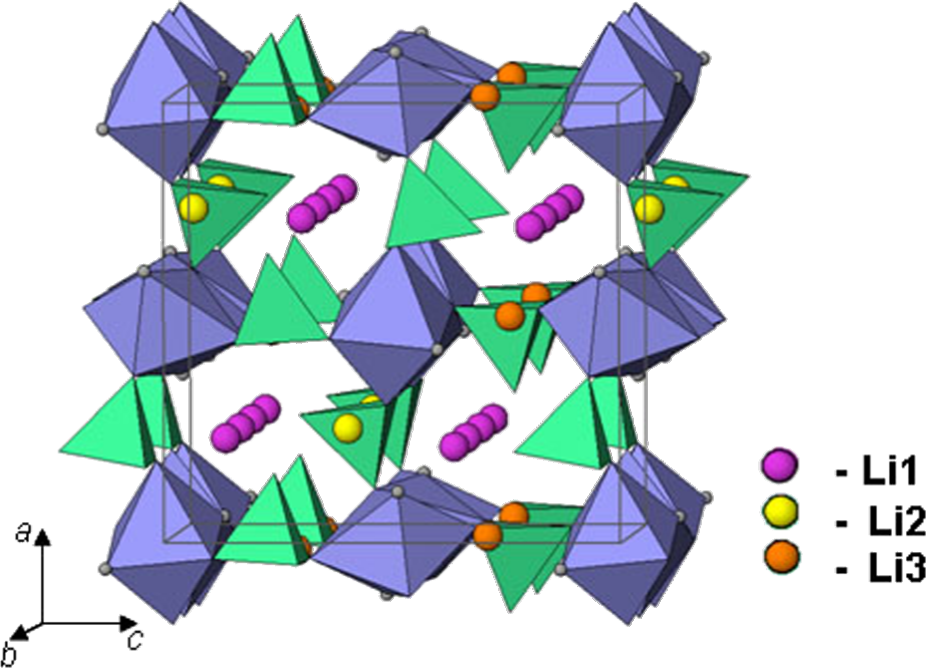
Crystal structure of 3D-Li_2_MPO_4_F, positions of Li atoms are denoted.

Li_2_CoPO_4_F, which exhibits an electrochemical activity above 5 V vs Li/Li^+^, is one of the attractive candidates in the fluorophosphate family [1]. This fluorophosphate possesses a three-dimensional (3D) tunnel structure and, by analogy to the olivine phase, is expected to demonstrate a good stability and reversibility upon cycling. It is built of edge-shared CoO_4_F_2_-octahedra interconnected with PO_4_-tetrahedra, which generate a framework with channels through which alkali-ion diffusion can take place [2–4] (Figure 1). The reversible electrochemical activity of Li_2_CoPO_4_F has been studied by several groups [4–9]. Our previous investigation of this cathode material has revealed the de/intercalation of lithium occurs through a single-phase reaction mechanism. Moreover, according to the capacity–voltage dependence the extraction of more than one Li^+^ ion should take place at potentials larger than 5.5 V [4], which is beyond the stability range of conventional electrolytes. An initial discharge capacity of 132 mA·h·g^−1^ that is delivered by Li_2_CoPO_4_F in a high-voltage electrolyte with fluorinated alkyl carbonates has been reported by S. Amaresh et al., however noticeable capacity fading has been observed upon prolonged cycling [8]. Therefore, the evaluation of the electrochemical performance of Li_2_CoPO_4_F and the other representative of this family such as Li_2_NiPO_4_F [2,10], is limited to conventional electrolytes. Hence, the development of new organic electrolytes with a wide range of application voltages and the investigation of high-voltage fluorophosphates using these new electrolyte systems are strongly required.

Another way to explore this fluorophopshate family is to adjust the operating voltage of these compounds to values that are sustained by conventional electrolytes. This might be achieved through a complete or a partial substitution of Co^2+^ by Fe^2+^ or Mn^2+^ with lower values of the of M^2+^/M^3+^ redox potential. Here, we report on the synthesis and the investigation of Li_2_Co_1−_*_x_*M*_x_*PO_4_F (M = Fe, Mn) fluorophophates, which have not been yet identified. Furthermore, different high-voltage electrolytes systems were tested and utilized to evaluate the electrochemical performance of the new synthesized compounds.

## Results and Discussion

### Testing of electrolytes

An electrochemical window that extends above 5.5 V (vs Li/Li^+^) has been reported for several electrolytes systems based on sulfone or dinitrile solvents [10–14]. For instance, tetramethylene sulfone (TMS) in the presence of an imide salt (LiTFSI) demonstrated a resistance to electrochemical oxidation up to 6 V vs Li/Li^+^ [11], while 1 M LiBF_4_/(EC)/DMC/sebaconitrile was used to examine the high-voltage performance of the fluorophosphate Li_2_NiPO_4_F [10]. We chose 1 M LiBF_4_/TMS to investigate the electrochemical activity of the fluorophosphate materials. LiBF_4_ salt was chosen instead of LiTFSI, because the last one corrodes the aluminum current collector at high potentials.

Preliminarily, the stability of both electrolytes was investigated by cyclic voltammetry to further establish their compatibility with high-voltage cathode materials. Two types of working electrodes were used to evaluate the electrochemical window of the electrolytes: 1) Al-foil (since it is used as a current collector for the positive electrode); 2) an “idle electrode”, which consisted of Al_2_O_3_/C/PVdF in a ratio of 80/10/10, in order to imitate the effect of the carbon- and binding electrode components at high potentials. Because the loading mass and the effective surface area of the active material on the electrodes that were used for electrolyte testing were similar in all experiments, the obtained current values were compared without normalization.

**Figure 2:**
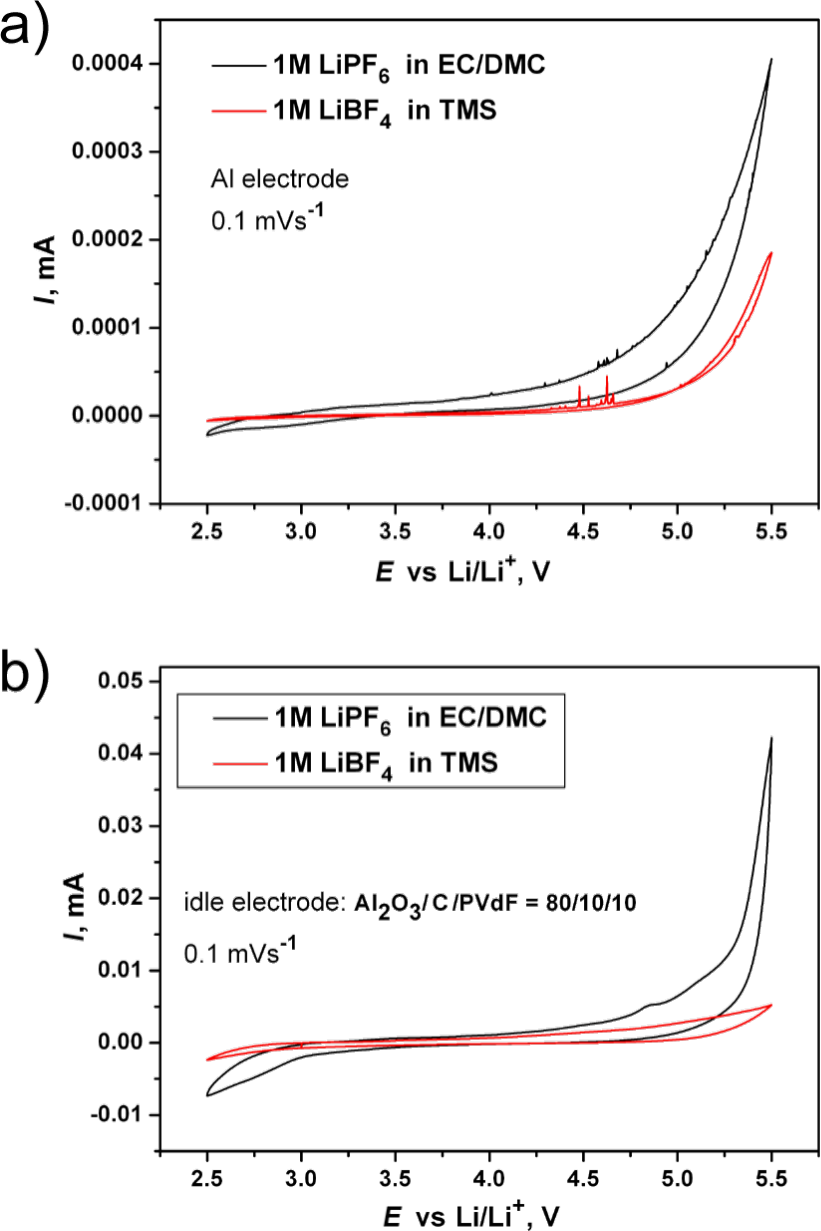
Cyclovoltammetry curves (first cycle) of 1 M LiPF_6_ in EC/DMC (black) and 1 M LiBF_4_ in TMS (red) at scan rate of 0.1 mV·s^−1^. (a) Aluminum electrode, (b) idle electrode consisting of Al_2_O_3_/C/PVdF in an 80/10/10 ratio.

Both electrolytes exhibited an electrochemical stability up to 5.5 V (vs Li/Li^+^) with aluminum as the working electrode (Figure 2a). For the first cycle the current detected at the highest potential did not exceed 0.4 μА, and it decreased (to 0.001 μА) upon subsequent cycling. It is clearly seen that the effect of the oxidation processes occurred at the Al electrode is negligible for both electrolytes when compared to a scanning with the idle electrode (Figure 2b). In the anodic sweep the commercial electrolyte showed a small increase in oxidation current at 4.8 V followed by drastic growth (up to 40 μA) around 5.2 V, while for the TMS electrolyte irreversible oxidation current peaks of 5 μA were detected. These results confirmed the reasonable stability of 1 M LiBF_4_/TMS electrolyte up to 5.5 V, which agrees with data reported previously [12–13].

### Investigation of Li_2_(Co,M)PO_4_F (M = Mn, Fe)

Applied synthesis approaches were directed not only towards the investigation of Li_2_Co_1−_*_x_*M*_x_*PO_4_F solid solutions, but also to the preparation of the corresponding electrode materials. Because of poor electronic and ionic conductivity that is inherent to polyanionic compounds, a carbon coating (for improving the electronic surface conductivity) and a downsizing of the particles (in order to shorten the Li-ion transfer paths) were applied to enhance the electrochemical performance of the investigated materials. In order to reduce the particle size and to prevent grain coalescence the lowest temperatures usable for the formation of the pure olivine precursors and the fluorophosphates were always chosen.

**Figure 3:**
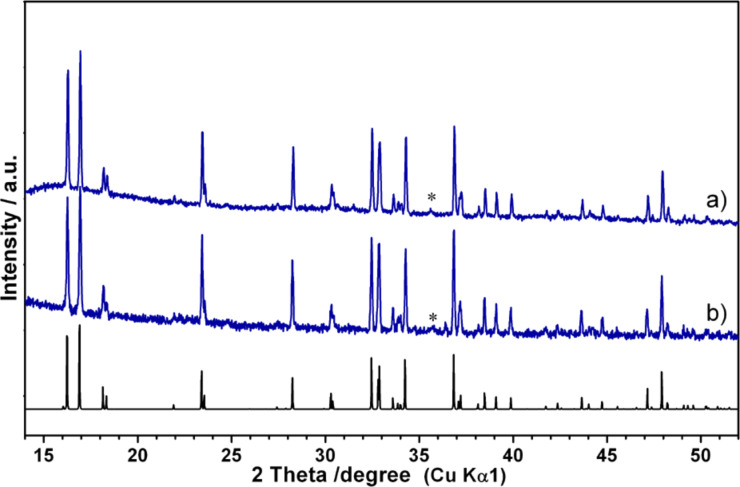
Powder XRD patterns of Li_2_CoPO_4_F/C (a) and Li_2_Co_0.9_Mn_0.1_PO_4_F (b). A theoretical pattern of Li_2_CoPO_4_F calculated by using PDF 56-149 is shown on the bottom. Reflections corresponding to WC are marked by an asterisk.

**Figure 4:**
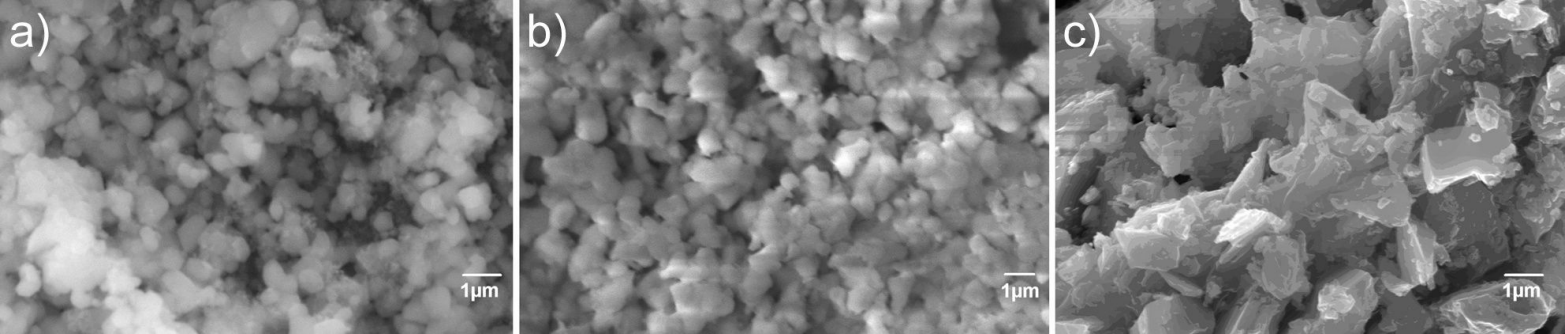
SEM images of fluorophosphate materials a) Li_2_CoPO_4_F/C, b) Li_2_Co_0.9_Mn_0.1_PO_4_F/C, c) Li_2_Co_0.7_Fe_0.3_PO_4_F.

The Li_2_CoPO_4_F/C composite for electrochemical measurements was synthesized according to a procedure that was optimized previously [4]. A mixture of LiCoPO_4_/C with 1.05 equiv of LiF was annealed at 670 °C for 1 h under Ar-flow and subsequently quenched to room temperature. The XRD pattern confirmed the formation of Li_2_CoPO_4_F, though a small amount of WC (about 1%, from the ball-milling media) was also detected (Figure 3). The refined unit cell parameters of Li_2_CoPO_4_F/C (*a* = 10.444(3) Å, *b* = 6.374(2) Å, *c* = 10.868(3) Å, *V* = 723.6(5) Å^3^) were in agreement with previously reported data [1,4]. The residual carbon in Li_2_CoPO_4_F/C was found to be 1.7%. According to the SEM images the synthesized material consisted of almost uniform particles with an average size of 0.7–0.9 μm (Figure 4).

In order to investigate the Li_2_Co_1−_*_x_*Mn*_x_*PO_4_F solid solutions a combination of freeze-drying and solid-state techniques was applied. A mixture of LiCo_0.9_Mn_0.1_PO_4_ obtained from cryogranulate was annealed with 1.05 equiv of LiF in the temperature range of 650–700 °C for 1–2 h. Annealing at 680 °C for 1 h was found to be optimal for the preparation of the Li_2_Co_0.9_Mn_0.1_PO_4_F phase. The XRD pattern of this sample (Figure 3) was indexed as an orthorhombic unit cell with parameters *a* = 10.465(2) Å, *b* = 6.3998(9) Å, *c* = 10.898(2) Å and V = 729.9(2) Å^3^. No peaks of the olivine phase were observed, though a small amount of WC (about 1%, from the ball-milling media) was detected in the XRD pattern. Further attempts to increase the Mn content in Li_2_Co_1−_*_x_*Mn*_x_*PO_4_F (*x* = 0.2, 0.3) by varying the annealing temperature and the heating duration ended up with multiphase samples that contained impurities of olivine and Li_3_PO_4_. Moreover, the unit cell parameters of the formed fluorophosphates were found to be close to those of Li_2_Co_0.9_Mn_0.1_PO_4_F. These results clearly indicated that Li_2_Co_1−_*_x_*Mn*_x_*PO_4_F exhibited a very limited range for the solid solution (*x* ≤ 0.10). For electrochemical testing Li_2_Co_0.9_Mn_0.1_PO_4_F/C was synthesized by adding carbon black (5 wt %) to the olivine precursor at an intermediate step of preparation. The XRD pattern of the obtained sample confirmed the formation of pure fluorophosphate with cell parameters similar to those given above. EDX analysis of the prepared material found the Co/Mn ratio to be 0.89(1)/0.11(1), which agreed with the expected values from the chemical formula. The morphology of this sample was investigated by SEM and showed particles of submicron size (Figure 4). The residual carbon in the prepared composite was determined to be as 3.1% by TG analysis. This value was taken into account during the preparation of the electrode.

**Figure 5:**
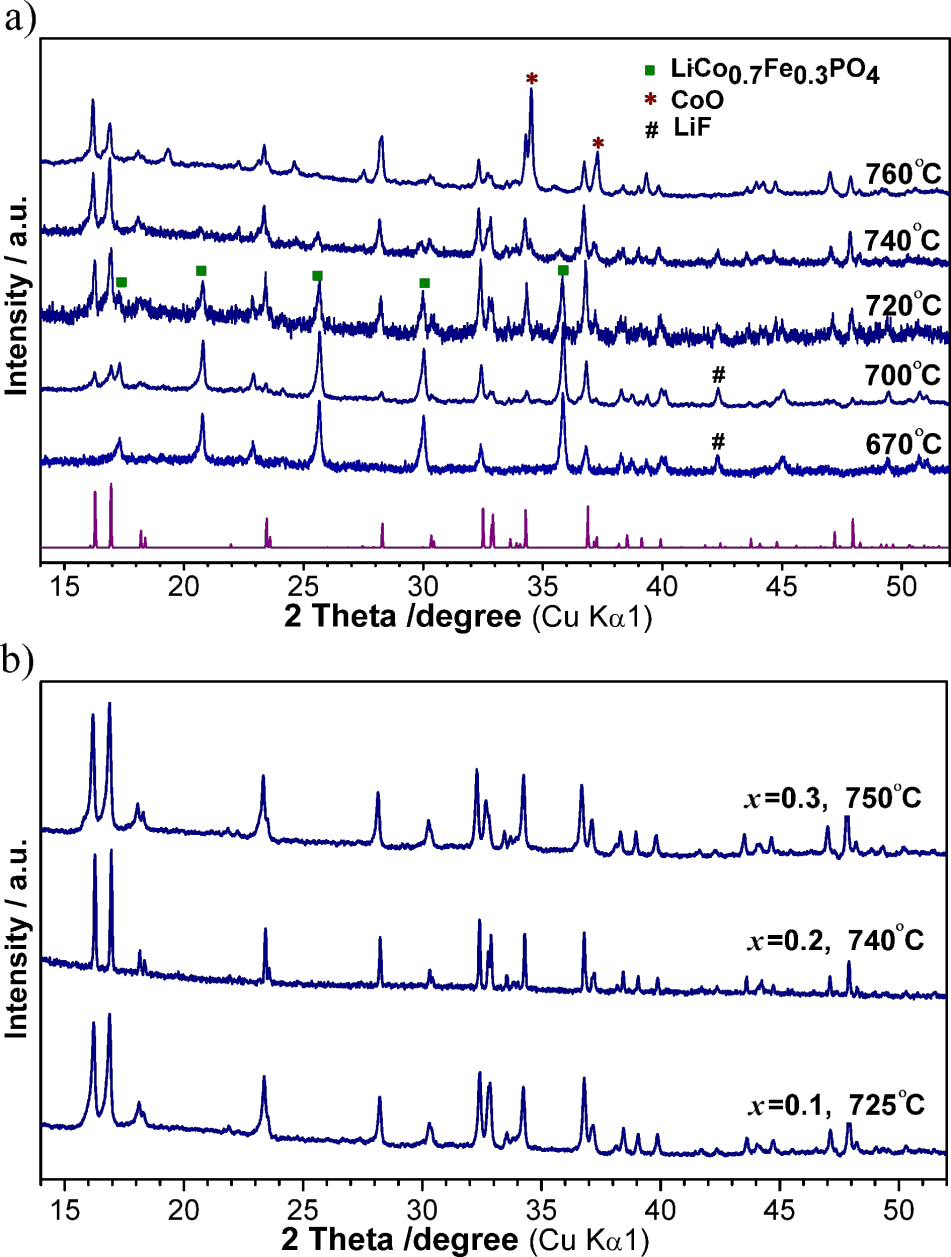
a) XRD patterns of a mixture of LiCo_0.7_Fe_0.3_PO_4_ and LiF, annealed at different temperatures, starting from 670 °С. XRD peaks that correspond to impurities are marked. b) XRD patterns of Li_2_Co_1−_*_x_*Fe*_x_*PO_4_F (*x* = 0.1, 0.2, 0.3), synthesized at the denoted temperatures.

The synthesis of the iron-substituted fluorophosphates, Li_2_Co_1−_*_x_*Fe*_x_*PO_4_F, was performed by a two-step solid-state process. The optimization of the preparation conditions was done for the composition of *x* = 0.3. Figure 5a represents XRD patterns of the samples obtained by annealing mixtures of LiCo_0.7_Fe_0.3_PO_4_ and LiF (with 10 wt % excess) at different temperatures. According to the XRD data, the fluorophosphate phase started to form above 700 °С, and further enhancement of the annealing temperature resulted in a decrease of the olivine impurities and in an increase of the fluorophosphate constituent. The formation of the almost pure Li_2_Co_0.7_Fe_0.3_PO_4_F was observed upon heating at 740–750 °С. Above these temperatures (>760 °С) samples melted and were heavily contaminated by cobalt oxide. Thus, the annealing at 750 °С for 1 h in Ar was found to be optimum to yield Li_2_Co_0.7_Fe_0.3_PO_4_F. A tuning of the annealing temperature allowed us to synthesize pure fluorophosphates with different levels of substitution, Li_2_Co_1−_*_x_*Fe*_x_*PO_4_F (*x* = 0.1–0.3) (Figure 5b). The XRD patterns of obtained samples were indexed on the base of an orthorhombic structure with a *Pnma* space group and the unit cell parameters that are listed in Table 1. Careful inspection of the XRD data revealed negligible amounts of Li_3_PO_4_ and Co admixtures. It is evident from the obtained results that the synthesis of Fe-substituted compounds requires increased annealing temperatures that depend on the Fe-content in Li_2_Co_1−_*_x_*Fe*_x_*PO_4_F. For a higher Fe-substitution higher annealing temperatures are needed. The solid-state synthesis at elevated temperatures resulted in large micrometer-sized particles (2–4 μm) as observed by SEM (Figure 4). It should be noted that the presence of LiF, which is used as the reagent, promoted the coalescence of small particles and induced crystallite growth because of fluxing at elevated temperatures. In spite of varying the preparation conditions all attempts to increase the substitution level of Fe in Li_2_Co_1−_*_x_*Fe*_x_*PO_4_F (*x* = 0.4, 0.5) led to multi-phase samples, with the fluorophosphate phases having cell parameters close to those of Li_2_Co_0.7_Fe_0.3_PO_4_F. Thus, it was concluded that the solid-solution range of Li_2_Co_1−_*_x_*Fe*_x_*PO_4_F was limited to *x* ≤ 0.3. Efforts to prepare a Li_2_Co_0.7_Fe_0.3_PO_4_F/C composite by adding carbon black or glucose to the initial mixtures of reagents resulted in multiphase samples that contained large amounts of metallic Co (>10%), which can be explained by the strongly reductive conditions that appeared at elevated temperatures (>700 °С) because of the presence of C-containing additives. Therefore, for the electrochemical evaluation of the Fe-substituted fluorophosphates the electrodes were prepared from the carbon-free product Li_2_Co_0.7_Fe_0.3_PO_4_F by thoroughly mixing it with Super-C carbon (10 wt %).

**Table 1:** Unit cell parameters of fluorophosphates Li_2_Co_1−_*_x_*M*_x_*PO_4_F (M = Mn, Fe).

*x* (M)	unit cell parameters of Li_2_Co_1−_*_x_*M*_x_*PO_4_F			

	*a*, Å	*b*, Å	*c*, Å	*V*, Å^3^

0	10.439(2)	6.3731(12)	10.864(2)	722.8(2)
0.1 (Mn)	10.465(2)	6.3998(9)	10.898(2)	729.9(2)
0.1 (Fe)	10.440(2)	6.3862(13)	10.867(3)	724.5(4)
0.2 (Fe)	10.442(2)	6.4103(14)	10.884(2)	728.6(3)
0.3 (Fe)	10.453(1)	6.4096(8)	10.888(12)	729.5(2)

According to the obtained results Li_2_Co_1−_*_x_*Fe*_x_*PO_4_F and Li_2_Co_1−_*_x_*Mn*_x_*PO_4_F systems exhibit limited ranges of solid solution. This finding might be explained by differences in the sizes of transition metal ions: Apparently, the structure framework becomes unstable upon higher substitution of Co^2+^ (0.735 Å) by larger Fe^2+^ (0.780 Å) and Mn^2+^ (0.820 Å) [15]. Indeed, while Li_2_MPO_4_F (M = Co, Ni) can be obtained by direct synthesis, the preparation of 3D-Li_2_FePO_4_F requires the electrochemical ion-exchange of the Na-counterpart, and the corresponding Mn-based fluorophosphate has not been yet identified [16]. It is reasonable, that a substitution of Co^2+^ by Mn^2+^, which has the largest ionic radius, only takes place in a smaller range (*x* ≤ 0.10) than in the case of Fe^2+^ (*x* ≤ 0.30). In both cases the substitution results in considerable expansion of the unit cell (ca. 7 Å^3^) for the highest level of substitution (Table 1).

### Electrochemical performance of Li_2_(Co,M)PO_4_F (M = Mn, Fe)

**Figure 6:**
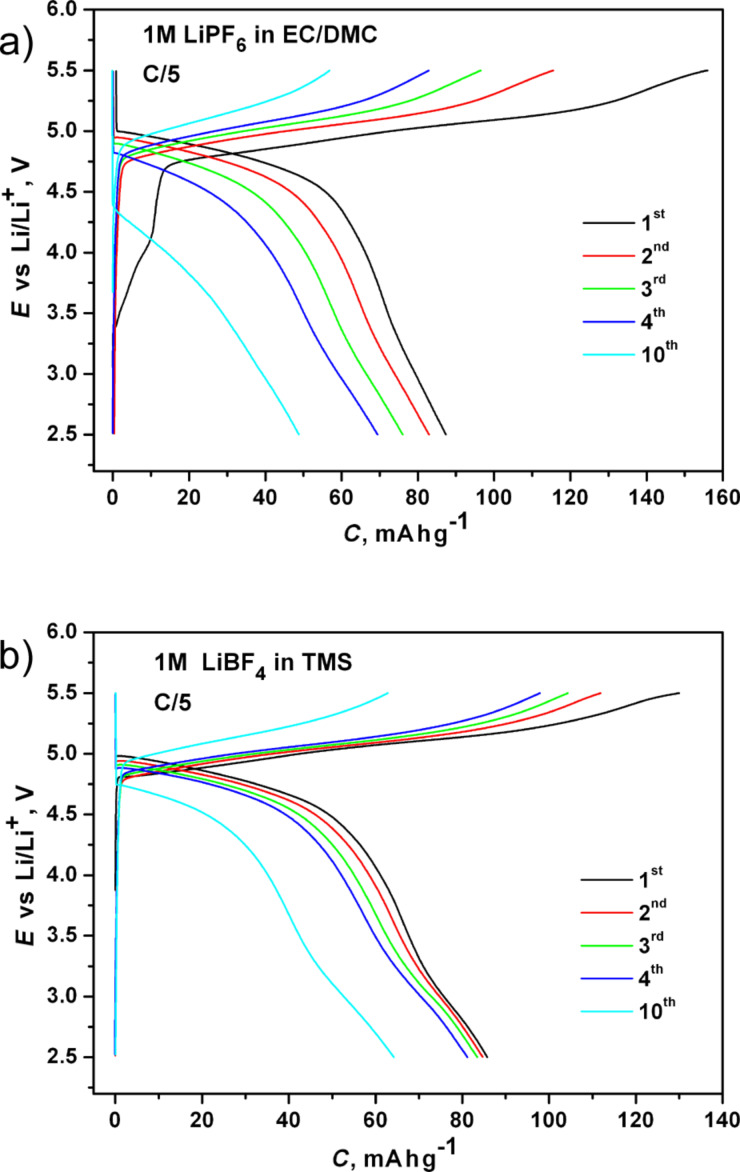
Charge-discharge curves of Li_2_CoPO_4_F in the commercial (a) and in the sulfone-based electrolytes (b) measured at C/5.

According to galvanostatic measurements performed at a rate of C/5 (Figure 6) Li_2_CoPO_4_F starts to discharge at approx. 5 V, which agrees well with previous results. The Li/Li_2_CoPO_4_F cells delivered initial discharge capacities of ca. 90 and 85 mA·h·g^−1^ with the commercial and the sulfone-based electrolyte, respectively, and these values corresponded to a reversible de/intercalation of about 0.65 Li. During the initial cycles the charge capacity values were remarkably higher than the corresponding discharge capacities. This discrepancy in the capacities may result from a decomposition of the electrolyte on the conductive carbon and on the flurophosphate material at high potentials. For the TMS electrolyte this discrepancy disappeared upon subsequent cycling. During the 10th cycle the corresponding values became almost equal, with a coulombic efficiency of 98% (Figure 6). Moreover, there is less capacity fading when using the TMS electrolyte. During the 10th cycle the discharge capacity decreased to about 83% of the initial value in contrast to a decrease to about 45% found with the commercial electrolyte. The obtained results indicated a rather stable electrochemical performance of the Li_2_CoPO_4_F material at high voltages in the 1 M LiBF_4_/TMS electrolyte. The decrease of the irreversible capacity, which leads to the high columbic efficiency, implies that this electrolyte forms a stable solid-electrolyte interface on the electrode surface, but this suggestion should be further investigated and confirmed.

A preliminary investigation of the electrochemical behavior of Li_2_Co_0.7_Fe_0.3_PO_4_F was carried out with electrodes prepared from the well crystallized sample. Potentiodynamic measurements in both electrolytes resulted in broad peaks on the anodic and cathodic branches with the discharge capacity values being lower than 10 mA·h·g^−1^. Because of the poor electrochemical activity, which is ascribed to the non-optimized morphology of the electrode material (particle size of 2–4 μm), any comparisons of Li_2_Co_0.7_Fe_0.3_PO_4_F with the unsubstituted material were unreasonable.

**Figure 7:**
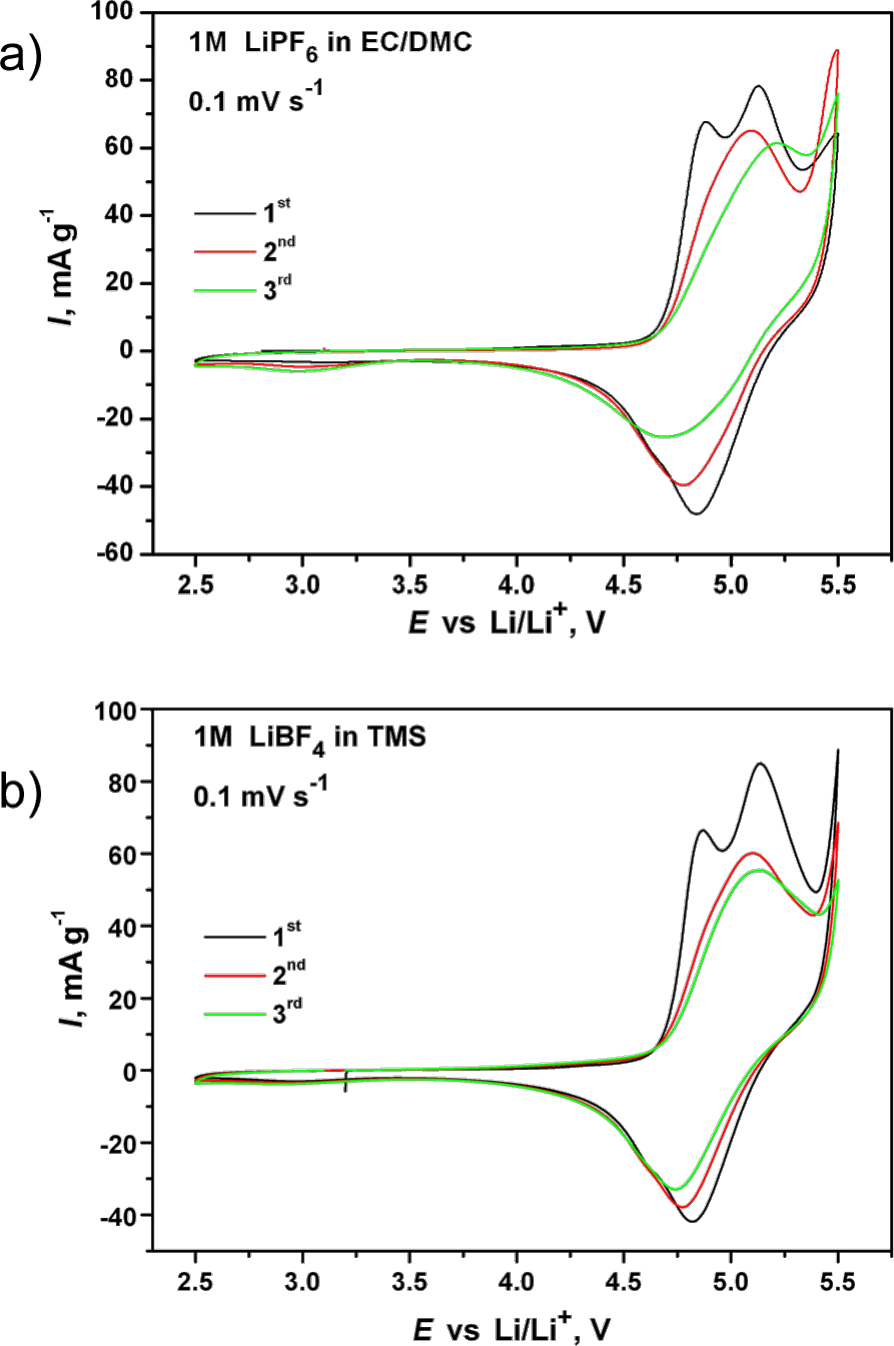
Cyclovoltammetry curves of the Li_2_Co_0.9_Mn_0.1_PO_4_F electrodes in the commercial (a) and the sulfone-based (b) electrolytes recorded at 0.1 mV s^−1^.

Figure 7 shows the cyclovoltammetry (CV) curves of the Li/Li_2_Co_0.9_Mn_0.1_PO_4_F cells cycled in both electrolytes. For the TMS electrolyte two oxidative peaks (at 4.9 V and 5.2 V) and a broad reductive peak (at 4.8 V) were observed in the first anodic and cathodic scans, respectively. During the second cycle the two oxidative peaks merged, and the broad peaks on the anodic (≈5.1 V) and cathodic (4.8 V) branches showed charge and discharge capacities of 135 and 70 mA·h·g^−1^, respectively. The CV curves that were recorded in the commercial electrolyte were quite similar. The presence of two oxidative peaks in the first anodic scan (Figure 7) hints at the occurrence of at least two redox processes. We related them to the structure transformation upon deintercalation of Li, followed by a further removal of Li from the transformed structure. This irreversible structure transformation, which occurs upon first charging, was investigated by ex-situ XRD studies and described in detail in our previous paper. This transformation resulted in an expansion of the framework and a probable redistribution of Li ions within the framework [4]. Similar features were observed in CV curves of Li_2_CoPO_4_F by D. Wang et al. [5] and S. Amaresh et al. [8]. This indicates the intrinsic nature of this transformation.

**Figure 8:**
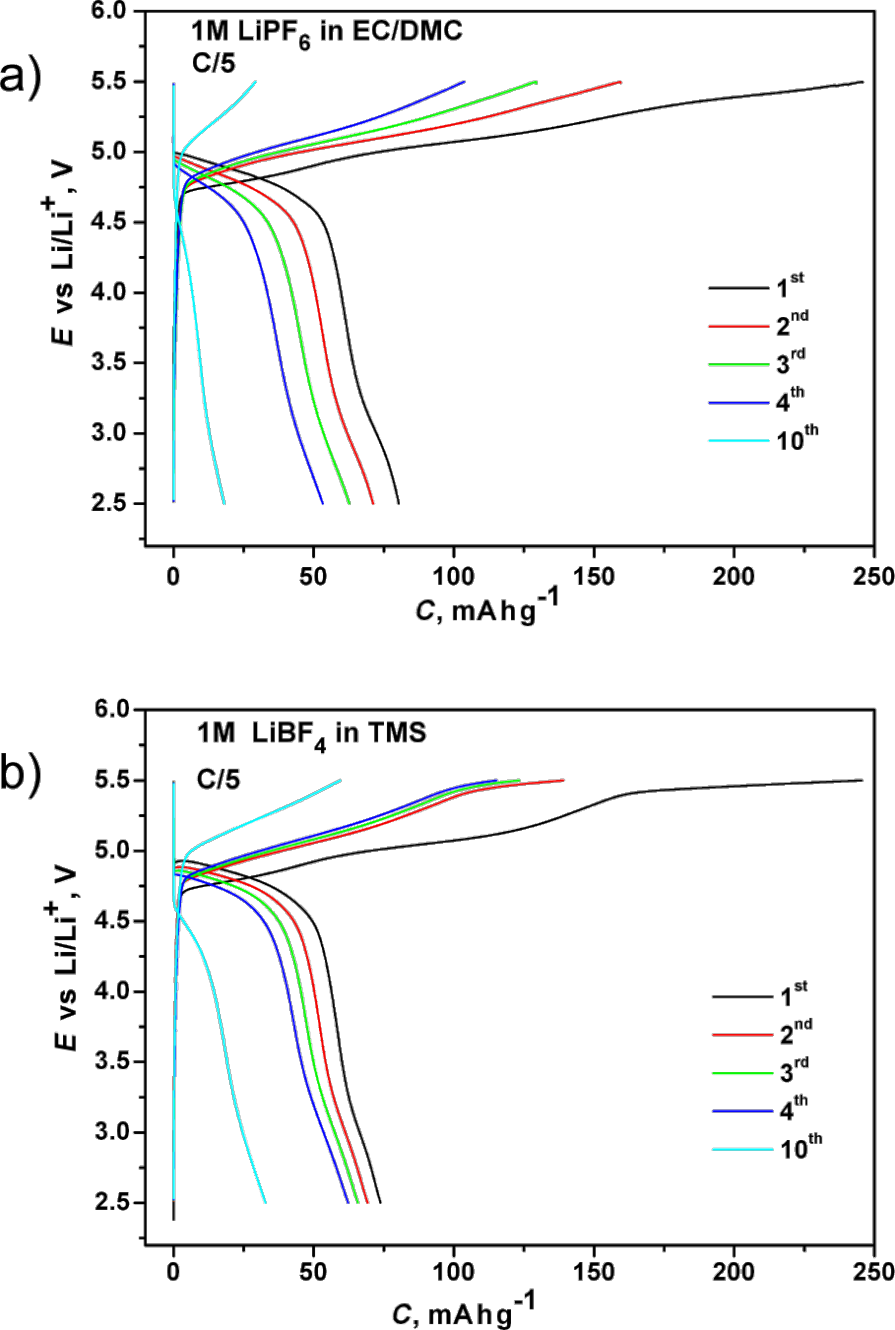
Charge–discharge curves of Li_2_Co_0.9_Mn_0.1_PO_4_/C in the commercial (a) and the sulfone-based (b) electrolytes measured at C/5.

Galvanostatic measurements on Li_2_Co_0.9_Mn_0.1_PO_4_F (Figure 8) revealed the highest discharge capacities of 75 and 85 mA·h·g^−1^ in TMS and the commercial electrolytes, respectively. As in the case of Li_2_CoPO_4_F, the capacity fading of the Mn-substituted fluorophosphate was slower in the TMS electrolyte. Sloping charge–discharge profiles and broad CV peaks suggest a single-phase (solid-solution) reaction mechanism, similar to Li_2_CoPO_4_F [4,8]. There is no visible change in the operating potential of Li_2_Co_0.9_Mn_0.1_PO_4_F. Therefore it was difficult to draw a decisive conclusion on the effect of Mn-substitution on the electrochemical activity of the Li_2_CoPO_4_F system. A further optimization in synthesis and formulation of the cathode material (particle investigation and carbon coating) of Mn- and Fe-substituted fluorophosphates will improve their electrochemical performance and, thereby, answer the question about a possible fine tuning of the operating voltage of this fluorophosphate family through substitutions on the transition metal site.

## Conclusion

New fluorophosphates, Li_2_Co_1−_*_x_*Mn*_x_*PO_4_F and Li_2_Co_1−_*_x_*Fe*_x_*PO_4_F, were successfully synthesized and investigated. Both systems exhibited narrow ranges of solid solution that agreed well with the ionic sizes of the transition metals. Good cycling and capacity behavior was attained with the 1 M LiBF_4_/TMS electrolyte. Galvanostatic measurements revealed a reversible electrochemical activity with discharge capacities as high as 90 and 75 mA·h·g^−1^ for Li_2_CoPO_4_F and Li_2_Co_0.9_Mn_0.1_PO_4_F respectively. A further investigation that includes the optimization of the electrode materials and the development of a high-voltage electrolyte is required to evaluate all potentials of this Li_2_Co_1−_*_x_*M*_x_*PO_4_F (M = Mn, Fe) fluorophosphate family.

## Experimental

The fluorophosphates, Li_2_CoPO_4_F and Li_2_Co_1−_*_x_*M*_x_*PO_4_F (M = Fe, Mn) were synthesized in a two-steps process. In the first step LiCo_1−_*_x_*M*_x_*PO_4_ olivine precursors were prepared through freeze-drying or ceramic techniques, depending on the transition metal. In the second step the obtained olivine samples were ball-milled with 1.05 equiv of LiF (5% excess), pelletized, annealed at 650–720 °C for 1–2 h under Ar flow and, subsequently, quenched to room temperature. As noticed above the technique applied to synthesize olivine precursors depended on the transition metal. Thus, LiCoPO_4_ was prepared by a solid-state reaction from a stoichiometric mixture of Li_2_CO_3_ (99.1%), (NH_4_)H_2_PO_4_ (99%), Co(NO_3_)_2_·6H_2_O (99.9%). It should be noted that the purity of initial reagents was checked by X-ray diffraction, the weight form of the crystallohydrates that were used for sample preparation was verified by thermogravimetric analysis. The initial reagents were mixed by planetary ball-milling, pelletized and then annealed in a tubular furnace (under steady Ar flow) at 380 °C for 10 h and at 600 °C for 15 h with intermediate regrinding. The Fe-substituted olivine precursors, LiCo_1−_*_x_*Fe*_x_*PO_4_, were obtained from stoichiometric mixtures of Li_2_CO_3_, NH_4_H_2_PO_4_, FeC_2_O_4_·2H_2_O (99%) and CoC_2_O_4_·2H_2_O (99%). The annealing profile was similar to that described for LiCoPO_4_. The oxalates, FeC_2_O_4_·2H_2_O and CoC_2_O_4_·2H_2_O, were chosen as initial reagents because the mixture of CO and CO_2_ released upon their decomposition suppressed the Fe^2+^ oxidation (in contrast to NO_2_ evolved by nitrates) and, at the same time, did not reduce Co^2+^ to metallic Co.

The Mn-substituted olivine precursors, LiCo_1−_*_x_*Mn*_x_*PO_4_, were prepared through the freeze spraying technique. The initial reagents LiCH_3_COO (99%), NH_4_H_2_PO_4_, Co(NO_3_)_2_·6H_2_O and Mn(CH_3_COO)_2_·3.2H_2_O were dissolved in distilled water and combined to form a transparent solution with a pH value of 3.0–3.5, which was adjusted by adding 1 M CH_3_COOH. This solution was exposed to freeze-spraying in liquid nitrogen, and the obtained product was subjected to vacuum sublimation in a Labconco sublimator (pressure 0.2 mbar, temperature range −40 to +30 °C) for 70 h. The prepared granulate was pressed into pellets and annealed at 350 °C for 10 h and at 550 °C for 15 h under Ar flow with intermediate regrinding.

The carbon-containing composites, Li_2_Co_1−_*_x_*M*_x_*PO_4_F/C, were prepared by adding carbon black (3–5 wt %) to the initial mixtures or, as in the case of M = Mn, to the products obtained from cryogranulates by annealing at 350 °C. The amount of residual carbon in the obtained composites was determined by thermal analysis and taken into account during the preparation of the electrodes.

Mechanical grindings (180–200 rpm for 2–3 h) were carried out in a Fritsch planetary micro-mill Pulverisette 7 while using a WC bowl, ZrO_2_ balls and acetone media. Thermal analysis was performed in the temperature range of 20–750 °C (10 °C/min heating rate) by using a thermo-gravimetric differential scanning calorimetry (TG-DSC) apparatus STA-449 (Netzsch, Germany).

The samples were characterized by powder X-ray diffraction (XRD) using a Huber G670 Guinier camera (Cu Kα_1_ radiation, Ge monochromator, image plate detector) and Bruker D8 Advance with a Lynxeye detector (Cu Kα radiation). The quantitative phase analysis for the selected samples was carried out by Rietveld refinement with the program JANA 2006 [17]. SEM investigation of powdered samples was performed with a JEOL JSM-6490LV scanning electron microscope equipped with an energy dispersive X-ray spectroscopy (EDX) attachment.

The electrochemical evaluation was performed in two-electrode-configuration cells with Li-metal foil acting both as the reference and counter electrodes, borosilicate glass was used as a separator. The positive electrodes were prepared by thoroughly mixing the active material (80 wt %) with carbon Timcal Super C (10 wt %) and PVdF (10 wt %) dissolved in a minimal amount of *N*-methyl-pyrrolidone. This cathode slurry was cast on an Al-foil collector by using the doctor-blade technique with a typical loading of 1 mg·cm^−2^. The prepared electrodes were dried, rolled and then dried again at 100 °C under vacuum for several hours. The electrochemical evaluation was carried out by using the following electrolytes: 1) 1 М LiPF_6_ solution in ethylene carbonate (EC) and dimethylcarbonate (DMC) with a volume ratio of 1:1 (commercial electrolyte, Merck); 2) 1 M solution of LiBF_4_ in tetramethylene sulfone (TMS). The latter electrolyte was prepared by dissolving an appropriate amount of LiBF_4_ (99.99%, Aldrich) in TMS that was purified up to 99.8% before. The electrochemical cells were assembled in an Ar-filled glove box. All tested cells were left to relax before the measurements (10–20 h). A potentiostat/galvanostat Biologic VMP-3 was used for data collecting. The cyclic voltammetry scanning was performed in the voltage range of 2.5–5.5 V at a scan rate of 0.1 mV·s^−1^. The galvanostatic charge–discharge cycling was conducted in the voltage range of 2.5–5.5 V at a rate of C/5 (the current required to deintercalate one Li ion from Li_2_Co_1-_*_x_*M*_x_*PO_4_F in 5 hours).

## References

[1] Okada S, Ueno M, Uebou Y, Yamaki J-i (2005). J Power Sources.

[2] Dutreilh M, Chevalier C, El-Ghozzi M, Avignant D (1999). J Solid State Chem.

[3] Hadermann J, Abakumov A M, Turner S, Hafideddine Z, Khasanova N R, Antipov E V, Van Tendeloo G (2011). Chem Mater.

[4] Khasanova N R, Gavrilov A N, Antipov E V, Bramnik K G, Hibst H (2011). J Power Sources.

[5] Wang D, Xiao J, Xua W, Nie Z, Wang C, Graff G, Zhang J-G (2011). J Power Sources.

[6] Dumont-Botto E, Bourbon C, Patoux S, Rozier P, Dolle M (2011). J Power Sources.

[7] Wu X, Gong Z, Tan S, Yang Y (2012). J Power Sources.

[8] Amaresh S, Kim G J, Karthikeyan K, Aravindan V, Chung K Y, Choc B W, Lee Y S (2012). Phys Chem Chem Phys.

[9] Kosova N V, Devyatkina E T, Slobodyuk A B (2012). Solid State Ionics.

[10] Nagahama M, Hasegawa N, Okada S (2010). J Electrochem Soc.

[11] Xu K (2004). Chem Rev.

[12] Abouimrane A, Whitfield P S, Niketic S, Davidson I J (2007). J Power Sources.

[13] Watanabe Y, Kinoshita S-i, Wada S, Hoshino K, Morimoto H, Tobishima S-i (2008). J Power Sources.

[14] Abu-Lebdeh Y, Davidson I (2009). J Electrochem Soc.

[15] Shannon R D (1976). Acta Crystallogr, Sect A.

[16] Khasanova N R, Drozhzhin O A, Storozhilova D A, Delmas C, Antipov E V (2012). Chem Mater.

[R17] 17Petricek, V.; Dusek, M.; Palatinus, L. *JANA 2006. The crystallographic computing system;* Institute of Physics: Praha, Czech Republic, 2006.

